# Transition to Adulthood Program: Indicated Prevention and Reduction of Time-to-Treatment in Adolescent Mental Health

**DOI:** 10.1192/j.eurpsy.2026.11143

**Published:** 2026-07-31

**Authors:** L. López Gómez-Miguel, Á. Privado Aranda, P. Bang Carrascosa, J. García Iglesias, A. Gutiérrez Fernández de Velasco, M. I. Montañés Rodríguez, M. González San José, A. Privado Aranda, A. Ladera de la Oliva, E. García Martín de la Fuente, L. M. F. Oliva, R. C. Ramos, M. D. R. D. F. Rodríguez, L. P. Ayuso Morales, M. Andrade Arango

**Affiliations:** 1 Programa de Transición y Primeros Consumos; 2 Hospital Universitario de Toledo, Toledo, Spain; 3 Universidad del Rosario, Bogotá, Colombia

## Abstract

**Introduction:**

Delays in accessing treatment for psychosis and other severe mental disorders can reach 8–10 years. Inspired by McGorry’s Australian model, the Transition to Adulthood Program in Toledo applies a targeted and cost-effective screening strategy based on two risk markers: age (16–20 years) and substance use.

**Objectives:**

- To describe the program design and clinical framework.
- To evaluate its ability to detect severe psychopathology at early stages.
- To analyze its impact on reducing treatment delays and improving service organization.

**Methods:**

Open-access program (2015–2025) including 1,300 patients. Inclusion criteria: adolescents/young adults (16–20 years) and/or substance or behavioral addictions. Multidisciplinary intervention (psychiatrists, psychologists, mental health nurses, social workers). Combined individual, group, and family psychotherapy, with daily open consultation slots.

**Results:**

- Two-thirds presented substance use; one-third met only age criteria.
- Mean age: 16.8 years; 60% female.
- 40% reported complex trauma history.
- 15% showed psychotic symptoms at intake; ~50% progressed to schizophrenia or bipolar disorder.
- ~400 patients treated annually; mean treatment duration: 18 months; 30% early dropout rate.

**Image 1:**
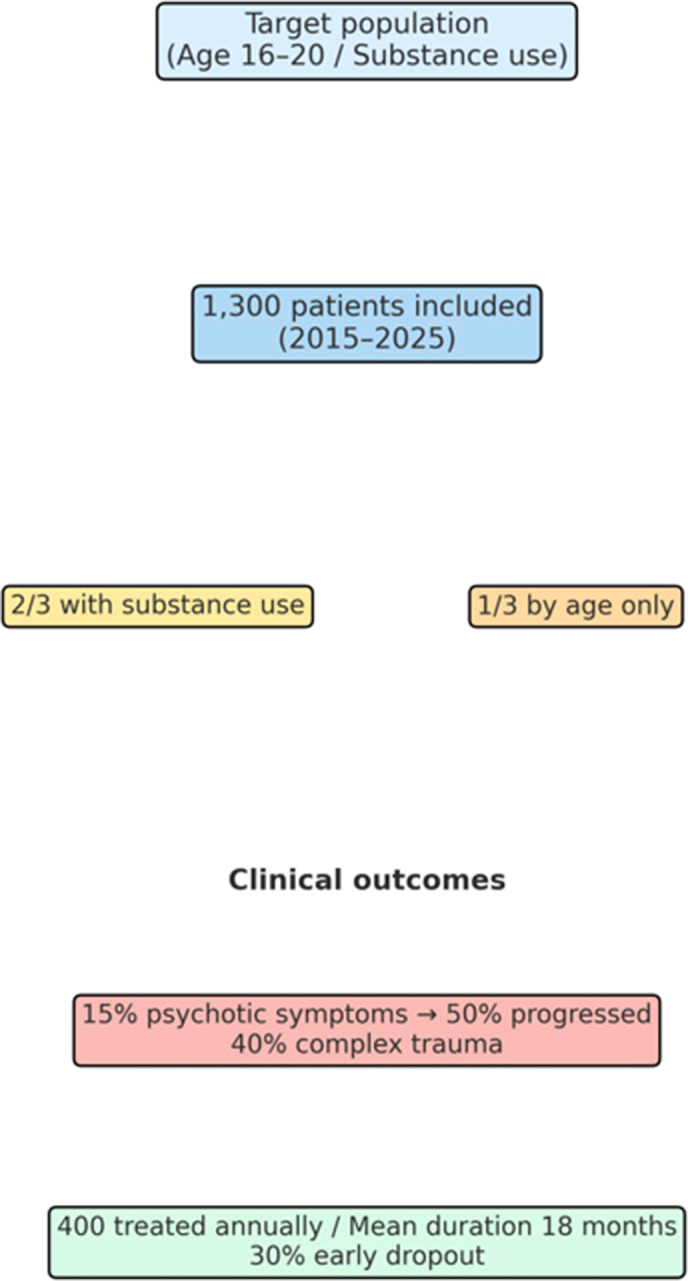

**Conclusions:**

The program achieved cost-effective early detection in high-risk populations, reduced time-to-treatment, and provided flexible, accessible care. This model demonstrates both clinical and organizational utility and can be adapted to other public mental health contexts.

**Disclosure of Interest:**

None Declared

